# Obstructive Pyelonephritis With Enterococcal Bacteremia Despite Unremarkable Urinalysis

**DOI:** 10.7759/cureus.102015

**Published:** 2026-01-21

**Authors:** Yoshihiro Kawaguchi, Sota Umetani, Kazuki Nitahata, Makoto Nakiri, Tsukasa Igawa

**Affiliations:** 1 Department of Urology, Saiseikai Futsukaichi Hospital, Fukuoka, JPN; 2 Department of Gastroenterology, Saiseikai Futsukaichi Hospital, Fukuoka, JPN; 3 Department of Anaesthesiology, Saiseikai Futsukaichi Hospital, Fukuoka, JPN; 4 Department of Urology, Kurume University School of Medicine, Kurume, JPN

**Keywords:** antimicrobial therapy, bacteremia, enterococcus faecalis, obstructive pyelonephritis, urinary tract obstruction

## Abstract

Obstructive pyelonephritis can be diagnostically challenging when urinary findings are unremarkable. *Enterococcus faecalis* is a well-known cause of complicated urinary tract infections and bacteremia; however, its clinical presentation may be atypical in patients with obstruction. A 73-year-old man presented with fever and left flank pain. Urinalysis and urinary sediment examination on admission were normal, and a urine culture was not obtained during the initial evaluation. Two sets of blood cultures yielded *E. faecalis*. Non-contrast computed tomography revealed a 12-mm left ureteral stone with hydronephrosis, leading to a diagnosis of obstructive pyelonephritis. Ureteral stenting was promptly performed, and empirical cefmetazole therapy was initiated. Despite adequate urinary drainage, fever persisted. *E. faecalis* was also isolated from renal pelvic urine obtained at the time of stent placement. Based on the clinical course and antimicrobial susceptibility results, therapy was switched to oral amoxicillin/clavulanate, resulting in rapid defervescence. Renal function improved markedly, and the patient recovered without septic shock or disseminated intravascular coagulation. This case highlights the importance of considering obstructive pyelonephritis despite negative urinalysis findings and reassessing antimicrobial therapy when fever persists after urinary drainage.

## Introduction

Obstructive pyelonephritis is a severe form of complicated urinary tract infection that occurs when urinary flow is impaired by obstruction, allowing bacterial proliferation within the upper urinary tract [1,2]. Prompt recognition and early source control are essential, as delayed diagnosis may lead to bacteremia, septic shock, and other life-threatening complications.

Although acute pyelonephritis typically presents with abnormal urinalysis findings such as pyuria and bacteriuria, these findings may be minimal or absent in cases of significant ureteral obstruction [1,3]. In such situations, inflammatory cells may not adequately reach the urine because urinary flow is impeded, resulting in deceptively normal urinalysis results. This diagnostic pitfall is particularly relevant in elderly patients, who often present with atypical manifestations of infection [3].

*Enterococcus faecalis* is a recognized pathogen in complicated urinary tract infections and bloodstream infections, especially among older adults and patients with underlying urological conditions [4]. Enterococcal infections may exhibit reduced susceptibility to commonly used empirical antibiotics, potentially leading to persistent infection despite appropriate urinary drainage [5].

Therefore, in suspected obstructive pyelonephritis, imaging studies and blood cultures are essential for diagnosis, and persistent fever after decompression should prompt reassessment of antimicrobial therapy [1,2]. Here, we report a case of obstructive pyelonephritis with *E. faecalis* bacteremia despite unremarkable urinalysis findings, highlighting key diagnostic and therapeutic considerations.

## Case presentation

A 73-year-old man with a history of type 2 diabetes was transferred to our emergency department with a three-day history of persistent left flank pain and high-grade fever of up to 40°C, initially suspected to be diverticulitis. Although flank pain was present, he denied lower urinary tract symptoms such as dysuria or urinary frequency. On admission, his temperature was 39.7°C, while his blood pressure and heart rate were stable.

Laboratory testing revealed leukocytosis (14,700/μL), an elevated C-reactive protein level (13.33 mg/dL), and a procalcitonin level of 5.21 ng/mL, indicating a substantial inflammatory response (Table 1).

**Table 1:** Laboratory findings on admission Laboratory parameters obtained at the time of hospital admission, including measured values and reference ranges. Leukocytosis, elevated inflammatory markers, and acute kidney injury were observed, whereas urinalysis showed no evidence of pyuria. HPF, high-power field; WBC, white blood cell.

Parameter	Value on admission	Unit	Reference range
WBC count	14,700	/μL	3,300–8,600
C-reactive protein	13.33	mg/dL	<0.30
Procalcitonin	5.21	ng/mL	<0.05
Serum creatinine	2.17	mg/dL	0.65–1.07
Urinalysis: leukocytes	Negative (1–4 WBC/HPF)	–	0–4 WBC/HPF

Serum creatinine was elevated to 2.17 mg/dL, consistent with acute kidney injury. Urinalysis and urinary sediment examination showed no pyuria. Because urinary symptoms were absent, urinalysis findings were unremarkable, and the initial diagnostic focus was on gastrointestinal pathology, a urine culture was not obtained at admission.

**Figure 1:**
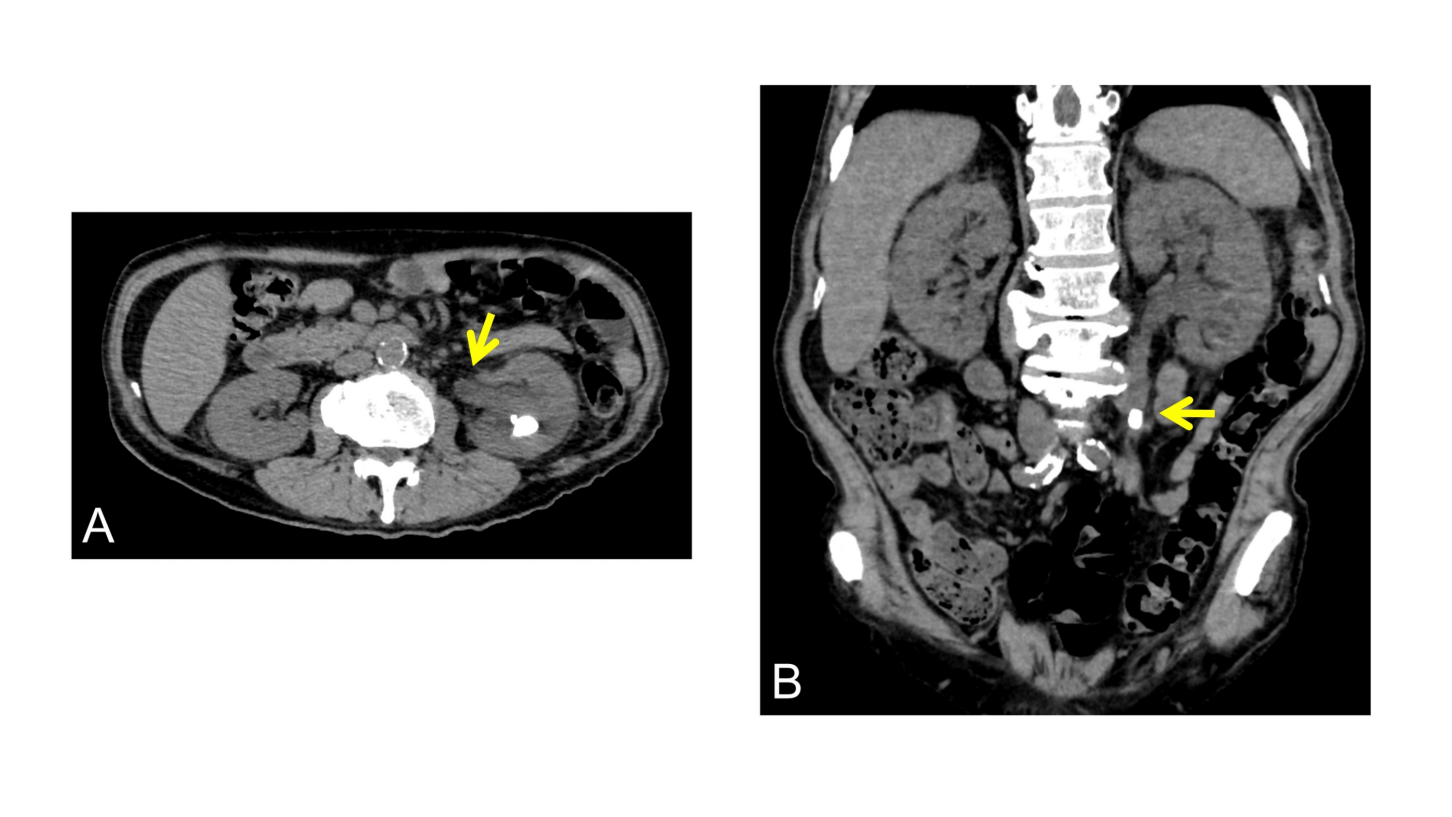
Computed tomography findings at presentation (A) Axial non-contrast computed tomography shows left hydronephrosis caused by a ureteral calculus (arrow). An intrarenal calculus is also present. (B) Coronal reconstructed image showing left hydronephrosis secondary to ureteral obstruction. The arrow indicates the causative ureteral stone.

Non-contrast computed tomography demonstrated a 12-mm calculus in the left ureter with associated hydronephrosis (Figure 1).

The patient was diagnosed with obstructive pyelonephritis and referred to the urology service. A left ureteral stent was placed for urinary drainage without lithotripsy, and empirical intravenous cefmetazole therapy was initiated. At the time of ureteral stent placement, approximately 25 mL of brownish urine was obtained from the left renal pelvis and submitted for culture. However, the fever persisted despite adequate urinary decompression.

During hospitalization, the patient did not develop septic shock, did not require vasopressor support, and showed no evidence of disseminated intravascular coagulation. Two sets of blood cultures obtained on admission grew *E. faecalis*, and the same organism was isolated from renal pelvic urine collected at the time of ureteral stent placement.

Transthoracic echocardiography performed on hospital day 7 revealed no valvular vegetations, making infective endocarditis unlikely. Antimicrobial susceptibility testing revealed susceptibility to ampicillin and amoxicillin/clavulanate. Given the persistent fever and the limited intrinsic activity of cefmetazole against enterococci, antibiotic therapy was switched to oral amoxicillin/clavulanate. The patient defervesced promptly, with marked improvement in his general condition. Renal function recovered significantly, with serum creatinine level decreasing to 1.08 mg/dL by hospital day 9.

**Figure 2:**
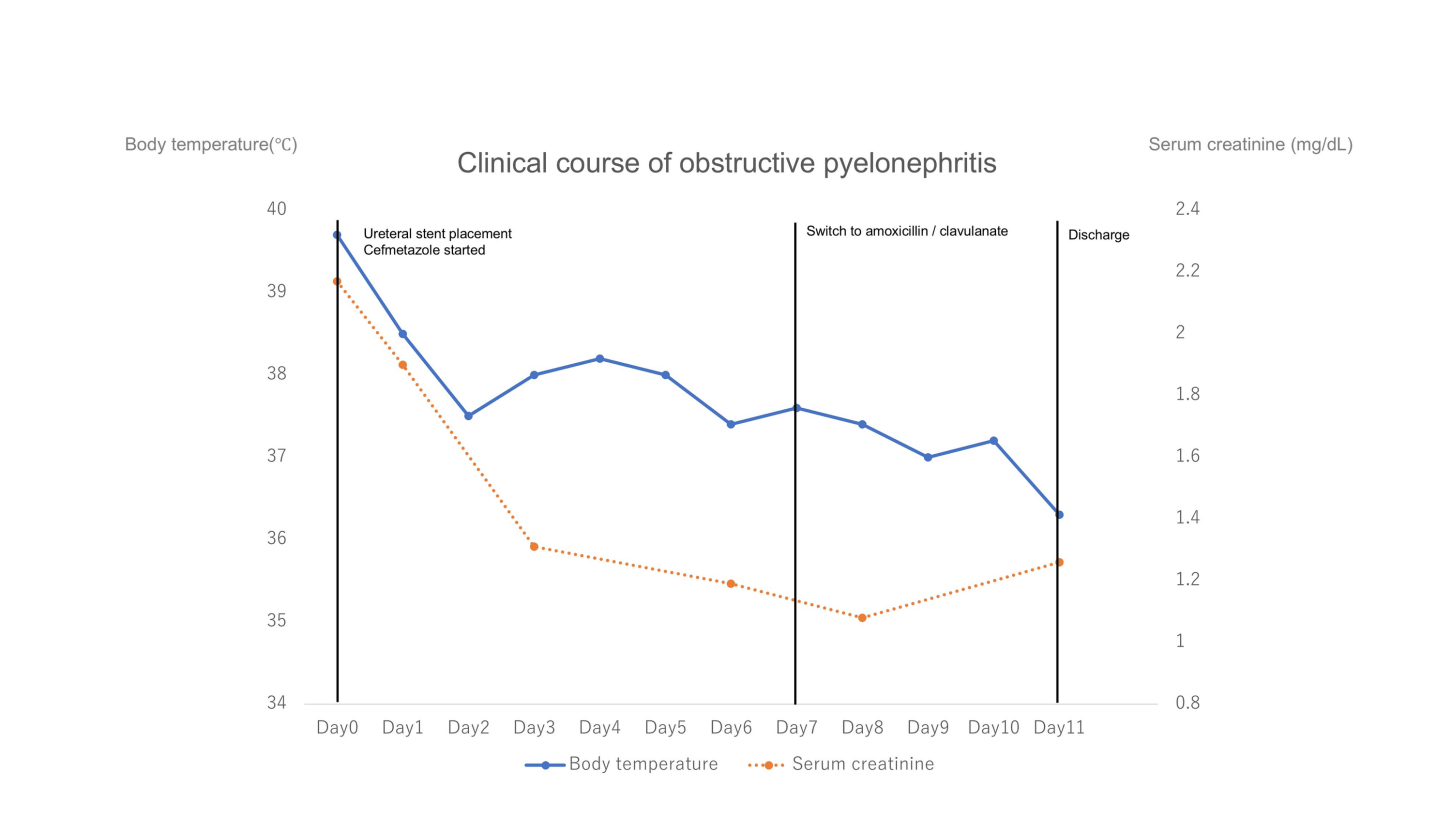
The patient’s clinical course Changes in body temperature and serum creatinine level during hospitalization are shown. A ureteral stent was placed on hospital day 0, and cefmetazole was initiated as empirical therapy. Because fever persisted despite adequate urinary drainage, antimicrobial therapy was switched to amoxicillin/clavulanate, after which defervescence and improvement in renal function were observed.

The patient completed the antibiotic course without complications and was discharged on hospital day 11 after confirmation of a negative procalcitonin level, despite the presence of mild residual low-grade fever without clinical deterioration (Figure 2).

## Discussion

From this case report, two important clinical lessons can be drawn. First, an upper urinary tract infection cannot be excluded solely based on negative urinalysis findings when significant urinary tract obstruction is present. Second, in obstructive pyelonephritis, persistent fever after adequate urinary drainage should prompt reassessment of antimicrobial therapy.

Enterococcal bacteremia often presents with non-specific clinical findings, and the urinary tract may be an underrecognized source of infection, particularly in elderly patients [4]. Obstructive pyelonephritis caused by urinary calculi is a well-established risk factor for systemic infection and bacteremia, as urinary stasis promotes bacterial proliferation and facilitates translocation into the bloodstream [1,2]. From a urological perspective, delayed diagnosis or insufficient source control in such cases substantially increases the risk of progression to urosepsis, underscoring the importance of early recognition and prompt intervention [6].

In elderly patients, urinary tract infections frequently present atypically, and classical findings such as pyuria or bacteriuria may be minimal or absent, even in the presence of active upper urinary tract infection [3]. Reliance on urinalysis alone may therefore result in diagnostic delay, particularly when urinary obstruction limits the flow of inflammatory cells into the urine. In such scenarios, imaging studies and blood cultures play a crucial role in establishing the diagnosis and identifying the causative pathogen.

*E. faecalis* is a common pathogen in complicated urinary tract infections and bloodstream infections among elderly patients and those with underlying urological conditions [4,7]. This organism possesses both intrinsic and acquired resistance mechanisms, including reduced susceptibility to commonly used β-lactam antibiotics, which may lead to persistent infection despite appropriate urinary drainage [5,8]. Current clinical guidelines emphasize that management of complicated urinary tract infections requires not only prompt urinary decompression but also timely initiation and adjustment of pathogen-directed antimicrobial therapy based on microbiological findings and clinical response [9,10].

In the present case, persistent low-grade fever despite successful urinary drainage highlighted the need to reassess antimicrobial therapy when clinical improvement was delayed. Adjustment of antibiotic treatment resulted in subsequent defervescence and clinical recovery, supporting the importance of ongoing clinical reassessment after source control. Furthermore, given the known association between enterococcal bacteremia and infective endocarditis, careful evaluation is warranted in such cases; transthoracic echocardiography performed on hospital day 7 revealed no evidence of valvular vegetations in this patient.

This report has several limitations. First, this is a single-case report, and the findings may not be generalizable to all patients with obstructive pyelonephritis. Second, a urine culture was not obtained at the initial presentation, which limits microbiological interpretation of the early disease course. Nevertheless, this case highlights important diagnostic and therapeutic considerations in obstructive pyelonephritis with enterococcal bacteremia and emphasizes the need for a comprehensive, multidisciplinary approach to management.

## Conclusions

This case demonstrates that obstructive pyelonephritis may be present despite normal urinalysis findings when significant urinary tract obstruction exists. Blood cultures and imaging studies played a critical role in establishing the diagnosis in this patient. In addition, persistent fever after adequate urinary drainage indicated the need to reassess antimicrobial therapy, which led to clinical improvement following antibiotic adjustment. Clinicians should maintain a high index of suspicion for upper urinary tract infection in similar presentations and carefully monitor the clinical response after source control to ensure optimal outcomes.
